# A Large Retrospective Study of Epidemiological Characteristics of COVID-19 Patients in the North of Iran: Association between SARS-CoV-2 RT-PCR Ct Values with Demographic Data

**DOI:** 10.1155/2022/1455708

**Published:** 2022-02-28

**Authors:** Farzin Sadeghi, Abazar Pournajaf, Mehrdad Halaji, Mohammad Chehrazi, Fatemeh Hejazi Amiri, Saghar Saber Amoli, Ali Hasanzadeh, Mostafa Javanian, Masoumeh Bayani, Mahmoud Sadeghi Haddad Zavareh, Mehran Shokri, Arefeh Babazadeh, Mohsen Mohammadi, Hamed Mehdinezhad, Mahmoud Monadi, Parviz Amri Maleh, Hamid Reza Nouri, Abdolreza Daraei, Mahdie Yousefnia Pasha, Mehdi Tourani, Seyed Raheleh Ahmadian, Nadia Esmailzadeh, Seyyedeh Maedeh Mirtabar, Shakiba Asadi, Ebrahim Nasiraie, Nafiseh Ezami, Shahrbano Gorjinejad, Kobra Fallhpour, Fatemeh Fakhraie, Yousef Beheshti, Mahnaz Baghershiroodi, Faeze Rasti, Maryam Salehi, Atiyeh Aleahmad, Rahman Babapour, Rahim Malekzadeh, Rahmat Habibzadeh Kashi, Yousef Yahyapour

**Affiliations:** ^1^Cellular and Molecular Biology Research Center, Health Research Institute, Babol University of Medical Sciences, Babol, Iran; ^2^Infectious Diseases and Tropical Medicine Research Center, Health Research Institute, Babol University of Medical Sciences, Babol, Iran; ^3^Department of Medical Microbiology, Faculty of Medicine, Babol University of Medical Sciences, Babol, Iran; ^4^Department of Biostatistics and Epidemiology, School of Public Health, Babol University of Medical Sciences, Babol, Iran; ^5^Department of Medical Microbiology, Faculty of Medicine, Golestan University of Medical Sciences, Gorgan, Iran; ^6^Non-Communicable Pediatric Diseases Research Center, Health Research Institute, Babol University of Medical Sciences, Babol, Iran; ^7^Department of Internal Medicine, Rouhani Hospital, Babol University of Medical Sciences, Babol, Iran; ^8^Department of Anesthesiology, Rouhani Hospital, Babol University of Medical Sciences, Babol, Iran; ^9^Department of Medical Genetics, Faculty of Medicine, Babol University of Medical Sciences, Babol, Iran; ^10^Babol Health Center, Babol University of Medical Sciences, Babol, Iran; ^11^Health Research Institute, Babol University of Medical Sciences, Babol, Iran; ^12^Part of Infectious Control, Rouhani Hospital, Babol University of Medical Sciences, Babol, Iran; ^13^Part of Medical Records, Rouhani Hospital, Babol University of Medical Sciences, Babol, Iran; ^14^Part of Infectious Control, Amirkola Hospital, Babol University of Medical Sciences, Babol, Iran; ^15^Part of Infectious Control, Shahid Beheshti Hospital, Babol University of Medical Sciences, Babol, Iran; ^16^Part of Infectious Control, Shahid Yahyanejad Hospital, Babol University of Medical Sciences, Babol, Iran; ^17^Genetics Laboratory, Shafizadeh Amirkola Children's Hospital, Babol University of Medical Sciences, Babol, Iran; ^18^Department of Clinical Biochemistry, Faculty of Medicine, Babol University of Medical Sciences, Babol, Iran

## Abstract

**Objectives:**

To avoid worsening from mild, moderate, and severe diseases and to reduce mortality, it is necessary to identify the subpopulation that is more vulnerable to the development of COVID-19 unfavorable consequences. This study aims to investigate the demographic information, prevalence rates of common comorbidities among negative and positive real-time reverse-transcriptase polymerase chain reaction (rRT-PCR) patients, and the association between SARS-CoV-2 cycle threshold (Ct) at hospital admission, demographic data, and outcomes of the patients in a large population in Northern Iran.

**Methods:**

This large retrospective cross-sectional study was performed from 7 March to 20 December 2020. Demographic data, including gender, age, underlying diseases, clinical outcomes, and Ct values, were obtained from 8,318 cases suspected of COVID-19, who were admitted to four teaching hospitals affiliated to Babol University of Medical Sciences (MUBABOL), in the north of Iran.

**Results:**

Since 7 March 2020, the data were collected from 8,318 cases suspected of COVID-19 (48.5% female and 51.5% male) with a mean age of 53 ± 25.3 years. Among 8,318 suspected COVID-19 patients, 3,250 (39.1%) had a positive rRT-PCR result; 1,632 (50.2%) patients were male and 335 (10.3%) patients died during their hospital stay. The distribution of positive rRT-PCR revealed that most patients (464 (75.7%)) had a Ct between 21 and 30 (Group B).

**Conclusion:**

Elderly patients, lower Ct, patients having at least one comorbidity, and male cases were significantly associated with increased risk for COVID-19-related mortality. Moreover, mortality was significantly higher in patients with diabetes, kidney disease, and respiratory disease.

## 1. Introduction

The new coronavirus disease 2019 (COVID-19) pandemic was firstly observed in late December 2019 which was caused by severe acute respiratory syndrome coronavirus 2 (SARS-CoV-2). It was firstly found in China, rapidly spreading to other Chinese provinces and other countries [1]. In general, COVID-19 results in asymptomatic, mild, or severe respiratory tract infections in human beings. It can result in a lethal condition in some cases [2]. Up to now, various studies were conducted on COVID-19, and multiple reports from different perspectives of disease were presented, including clinical manifestations, measures for treatment, and demographics of the disease [3]. It is known that there is an association between the risk of development of severe COVID-19 and some characterized individual conditions [4].

According to a previous study, the mortality rate ranges from 1.4% to 8% in the general population, and it increases significantly in patients with specific conditions and complications. Therefore, the risk of severe symptoms, even fatal conditions, and the poorer prognosis are higher in elderly adults and individuals with comorbidities [5–8]. A large number of hospitalized people, particularly patients that are eventually hospitalized in the intensive care unit (ICU) or lose their lives, experience comorbidities such as hypertension, chronic cardiovascular disease, obesity, and diabetes [9]. If the prevalence or frequency of these underlying diseases and related comorbidities are determined, it would be useful to gain a better knowledge of the prognosis of disease, the disease treatment, and the comprehensive management of outcomes [10].

SARS-CoV-2 viral genome detection is crucially important, and real-time reverse-transcriptase polymerase-chain-reaction (rRT-PCR) has served as a major and routine test to diagnose SARS-CoV-2 infection [11]. Several groups have suggested many viral targets to detect the virus, including nucleocapsid (N), open reading frame (ORF) 1a, RNA-dependent RNA polymerase (RdRp), spike (S), and envelope (E) [12]. The severity of COVID-19 may be worsened by the virus's main load and the quantity of virus a person possesses at any one moment. Viral load is a measure of the number of viral particles present in a person [13]. A person with high SARS-CoV-2 viral loads could get worse outcomes, and according to the data from China, the viral load is higher in patients with more severe diseases [13, 14]. The amount of virus exposure at the beginning of infection may increase the severity of the disease which is related to a higher viral load. However, as highlighted by recent studies, CT values cannot be directly affected by various factors, such as sample type, sampling time, assay design, and interpretation of the reports, and therefore must be interpreted with caution. Moreover, viral load in COVID-19 might be related to infectivity, disease phenotype, morbidity, and mortality [15, 16]. The cycle threshold (Ct) value obtained from a sample is an amplification measure needed for the target viral gene crossing a threshold value which can classify the viral genetic material's concentration approximately in a patient sample after rRT-PCR testing. This categorization is done as high, medium, or low. In the early phases of infection (before the capability of transmitting the infection by the patient) or late in infection when the risk of transmission is low, positive results with low viral load (high Ct) are observed. High infectivity and acute infection are presented by low Ct values (high viral load) [15, 17, 18]. Since, to date, few studies have investigated the relationships between SARS-CoV-2 viral load and mortality in a large patient cohort identification of the subpopulation with higher susceptibility to developing adverse outcomes of COVID-19, preventing the deterioration from moderate and mild to the severe conditions and reducing mortality are essential. Thus, the present study aims to investigate the demographic information, prevalence rates of common comorbidities among the negative and positive rRT-PCR patients, and the association between SARS-CoV-2 cycle threshold at hospital admission, demographic data, and outcomes of the patients in a large population in Northern Iran.

## 2. Methods

### 2.1. Patients and Study Design

This retrospective study was approved by the Ethics Committee of Babol University of Medical Sciences, Babol, Iran, with the ethics code IR.MUBABOL.REC.1400.012. Considering the retrospective nature of this study, the patients' written informed consent was waived by the committee.

This large retrospective cross-sectional study was performed between 7 March and 20 December 2020, and we collected the records of 8318 suspected COVID-19 patients who were admitted to hospitals (Ayatollah Rohani, Shahid Beheshti, Shahid Yahyanejad, and Amirkola Children Hospital) affiliated to Babol University of Medical Sciences (MUBABOL), in the north of Iran.

### 2.2. Data Collection

Patients' data were collected at the hospital centers using electronic medical records including demographic data, details of their medical history and comorbidities, underlying diseases, and clinical outcomes. We also collected the Ct value features for participants. Moreover, patients who did not have an oropharyngeal and nasopharyngeal swab sample, had unclear rRT-PCR results, or whose sample was analyzed on a different diagnostic platform or at a different institution were excluded.

### 2.3. Clinical Specimens

Oropharyngeal and nasopharyngeal samples were collected using flocked swabs from patients immediately after admission according to the World Health Organization (WHO) guidelines [19]. All samples were processed without further steps of dilution or heat inactivation according to standard laboratory biosafety guidelines. After processing, samples were divided into small volume aliquots and frozen at −80°C until the time of examination.

### 2.4. Viral Nucleic Acid Extraction and rRT-PCR for SARS-CoV-2 Detection

Laboratory confirmation of the SARS-CoV-2 was made using the rRT-PCR assay. Viral RNA was extracted from 300 *µ*L of swab sample storage media using the Ribospin vRD plus Kit (GeneAll, Seoul, South Korea) according to the manufacturer's instructions. After viral RNA extraction, rRT-PCR was used to detect the presence of SARS-CoV-2 using the LightMix® SarbecoV E-gene kit (Molbiol Germany) with LightCycler Multiplex RNA Virus Master (Roche) according to the manufacturers' protocol. A cycle threshold value of <36 Ct was defined as a positive test result. Sensitivity is 5.2 copies per reaction. The relative viral loads of their oropharynx and nasopharyngeal swab samples were estimated with Ct based on ABI One-Step rRT-PCR results. Patients were categorized based on diagnostic Ct values detected from the oropharyngeal and nasopharyngeal swabs that led to a SARS-CoV-2 infection diagnosis into the following three groups: Ct 10–20, Group A; Ct 21–30, Group B; and Ct 31–40, Group C.

### 2.5. Statistical Analysis

Categorical variables were summarized as frequencies and percentages. Continuous variables with a normal distribution were expressed as mean ± standard deviation (SD), and chi-square and Fisher's exact tests were used to perform intergroup and categorical comparisons as appropriate. Reported *p* values of <0.05 were considered statistically significant. SPSS software, version 16 (SPSS Inc., Chicago, IL, USA), was used to analyze the data.

## 3. Results

A total of 8318 suspected COVID-19 cases (48.5% female and 51.5% male) with a mean ± SD age of 53 ± 25.3 (range: 0 to 99) years were included in the study, of whom 4287 (51.5%) were male and 639 (7.7%) died of the disease during their hospital stay. Out of the 8318 suspected COVID-19 patients who were referred and hospitalized in our setting, 3250 (39.1%) had a positive rRT-PCR result; 1632 (50.2%) patients were male and 335 (10.3%) patients died during their hospital stay.

The details of positive and negative rRT-PCR results based on gender (*p*=0.053) and age (*p* ≤ 0.001) distribution and comorbidity are presented in Table 1. Based on age distribution, most of the suspected patients were ≥65 years (37.6%), while the lowest patients belonged to the group younger than one year (2.33%).

On the other hand, positive rRT-PCR rates increased with age, with a rate of 1% in patients aged <1 year, 2.6% in those aged 1–14 years, 2.1% in those aged 15–24 years, 20.8% in those aged 25–44 years, 38.6% in those aged 45–64 years, and 34.9% in those aged ≥65 years. Statistical analysis of age distribution showed that the occurrence of positive rRT-PCR was significantly increased with age, which is shown in Table 1.

Our data revealed that 61.3% of the study's suspected patients had one or more underlying conditions such as cardiovascular disease (CVD), diabetes, and brain and neurologic disorder. Accordingly, the most frequent comorbidity reported was CVD (2818/8318; 33.9%), followed by diabetes (2140/8318; 25.7%) and hypertension (1026/8318; 12.3%). On the other hand, the most prevalent comorbidities in patients with positive rRT-PCR were CVD (32%), diabetes (27.6%), and hypertension (13.8%), while 35.1%, 24.6%, and 11.4% of patients with negative rRT-PCR had CVD, diabetes, and hypertension. Statistical analysis of comorbidity distribution showed that comorbidities were significantly different in the two groups except for pregnancy (*p*=0.86), which are shown in Table 1.

Moreover, to better demonstrate the results related to Ct, the distribution of positive rRT-PCR revealed that 336 (10.4%) patients had a Ct between 10 and 20 (Group A), 2464 (75.7%) patients had a Ct between 21 and 30 (Group B), and 451 (13.9%) patients had a Ct between 31 and 40 (Group C).

Ct groups differed in terms of cardiovascular disease (*p*=0.06), hypertension (*p*=0.018), kidney diseases (*p*=0.001), malignancy (*p*=<0.001), and blood disorder (*p*=0.02); moreover, it seems that mortality was significantly different between the three groups, with the highest mortality in those with Ct between 10 and 20 (Group A = 16.7%) and lowest in the group with highest Ct (Group C = 8%) (*p* < 0.001). The Ct groups across age categories are shown in Table 2. Accordingly, it was noted that there was a statistically significant difference between increased age and the Ct groups.

There is a significant difference between the outcomes of patients across different age groups. Compared with discharged alive patients, death cases showed a significantly higher prevalence of comorbidities including kidney diseases (6.3% vs. 3.8%, *p*=0.031), diabetes (34.9% vs. 26.7%, *p*=0.001), and respiratory disorder (5.7% vs. 3%, *p*=0.008). The details of demographic information and comorbidities between discharged alive patients and death patients are shown in Table 3.

To evaluate the impact of gender in COVID-19, we compared the comorbidity and outcomes between male and female patients (Table 4). Results showed that the percentage of CVD (34.2% vs. 29.7%, *p*=0.006), diabetes (32% vs. 23.2%, *p*=<0.001), and hypertension (16% vs. 11.5%, *p*=<0.001) was significantly higher in female patients. Moreover, the mortality in male patients was significantly more (12.2% vs. 8.4%, in males and females, respectively, *p*=<0.001). Besides, 60.5% of the male patients had one or more comorbidities compared to female patients (60.5% vs. 52.7%, *p*=<0.001). The details of demographic information and comorbidities among female and male patients are shown in Table 4.

## 4. Discussion

In this cross-sectional study, we report the demographic, clinical, and outcome characteristics of 8,318 suspected COVID-19 patients who were admitted to the hospitals affiliated to Babol University of Medical Sciences (Babol, north of Iran) during the SARS-CoV-2 pandemic. Among collected samples, real-time reverse transcriptase PCR results showed that 39.1% (*n*: 3,250/8,318) were positive for SARS-CoV-2 genome, including 50.2% (*n*: 1,632/3,250) males and 49.8% (*n*: 1,618/3,250) females. There were no significant differences in positive rRT-PCR results between males and females (*p*=0.053). These data are in agreement with Goshayeshi et al. (2021) [20], Allameh et al. (2020) [21], and Trunfio et al. (2021) [22].

As shown in Table 1, the number of positive cases significantly increases with age (*p* < 0.001). Compared with the patients suspected of COVID-19, the discharge and death rates in patients with a definitive diagnosis of COVID-19 infection were 89.7% (*n*; 2,915/3,250) and 10.3% (*n*; 335/3,250), respectively, which is significant (*p* < 0.001). These results are consistent with Han et al. (2020) [23] and Bhaskaran et al. (2021) [24] who indicated that COVID-19 largely multiplies present risks faced by the patients, with some notable exceptions. Spanish researchers showed that aging is a potential risk factor for COVID-19 death. On the other hand, Azarkar et al. (2020) showed that the number of patients that are older than 60 is also positively associated with the death rate [25].Aging is independently associated with increased COVID-19 mortality, since our cases were mainly elderly with at least one comorbidity, including hypertension, CVD, diabetes, kidney diseases, respiratory disorder, malignancies, and cerebral, neurological, hepatogastrointestinal, and hematologic disorders. In our study, except for pregnancy, other comorbidities were significantly associated with death in the patients with COVID-19 (*p* < 0.05). In this regard, Mohammad Ebrahimi et al. reported malignancy and nervous and respiratory diseases to be significantly associated with increased case fatality rate. Moreover, in partial agreement with our results, their finding showed that diabetes, CVD, chronic renal diseases, nervous disease, and malignancy had higher rates in the nonsurviving group as compared with the surviving one [26].

Furthermore, in 2020, Davies et al. showed that the susceptibility to COVID-19 infection in individuals under 20 years of age is almost half that of adults over 20 years, and clinical symptoms have increased from 21% in people aged 10 to 19 years to 69% in people aged over 70 years [27]. In line with our data, Williamson et al. (2020) showed that the general cumulative prevalence of COVID-19 mortality rate 3 months after the beginning of the study was less than 0.01% in 18- to 39-year-olds, which increased to 0.67% and 0.44% in men and women over 80 years of age, respectively [28]. As you can see in Table 3 and in concordance with Davies et al. (2020), the death rate in SARS-CoV-2 infection is age-dependent [27]. In a study conducted in the UK, Davies et al. showed that age dependence in susceptibility to infection and the likelihood of having a clinically symptomatic exhibition of COVID-19 increased from ∼20% in children to ∼70% in the elderly. Therefore, there is evidence to show that there is both age-varying susceptibility to SARS-CoV-2 infection and age-varying severity in COVID-19 cases [27].

In concordance with our findings, Williamson et al. showed that the COVID-19-related deaths with a hazard ratio of 1.59 (95% CI 1.53–1.65) were associated with being male, being elderly, diabetes, severe asthma, and other medical complications [28]. Moreover, in case series from China, Europe, and the USA, COVID-19 hospitalizations, admission to ICU, invasive mechanical ventilation (IMV), and in-hospital deaths have consistently been higher in men than in women [29–34]. The reasons for gender differences in the consequences of COVID-19 can be as follows: (i) lifestyle differences, such as smoking, which is more common in men than in women and increases the risk of pneumonia and secondary infections after COVID-19; (ii) compared to men, the innate and acquired immune system is stronger in women; and (iii) a female sex hormone, estrogen, plays a protective role by activating the immune response and suppressing straight SARS-CoV replication [35–37]. The distribution of patients based on Ct of rRT-PCR was as follows: 10.4% with Ct 10–20 (Group A), 75.7% with Ct 21–30 (Group B), and 13% with Ct 31–40 (Group C).

Another interesting finding of this research was the existence of a substantial connection between Ct and both age and in-hospital mortality rate. In agreement with our data, Choudhuri et al. (2020) showed that the SARS-CoV-2 cycle threshold at admission was found to be an independent predictor of inpatient mortality [38]. According to the value of Ct, the highest and lowest mortality rates were related to Groups A and C, respectively. In concordance with our data, Dres et al. (2021) showed that the Ct values of RT-PCR were used as the indicators of the RNA viral load in the samples; the lower the Ct, the higher the RNA viral load [39]. We conclude that the lower the Ct, the higher the viral load, resulting in a more viral spread, severe illness, and death. However, Rabaan et al. (2021) stated that various factors could affect Ct values, including (i) preanalytic variables (such as collection technique, type of specimen, sampling time, and viral kinetics), (ii) analytic variables (for example, different kits and runs, internal control, type of RT-PCR, purity of reagents, and pipetting defects), and (iii) postanalytical variables (interpreting the reports) [40].

In a study conducted in New York, Magleby et al. (2019) showed that the hospital mortality rate was 35%, 18%, and 6% in the patients with high viral load (Ct < 25; *n* = 220), medium viral load (Ct 25–30; *n* = 216), and low viral load (Ct > 30; *n* = 242), respectively [41, 42].

Hence, the risk of IMV was 29%, 21%, and 15% in patients with a high, medium, and low viral load (*p* < 0.001). Zheng et al. (2020) [43] and Liu et al. (2020) [44] have described higher viral loads and longer persistence of the virus in patients with severe illness, as compared to those with mild infection. Contrary to the above studies, Shah et al. (2021) [41], Guan et al. (2020) [31], Argenziano et al. (2019) [45], and Zou et al. (2020) [46] have not found any relationship of Ct values with disease severity. Regarding the false-negative results in RT-PCR, Shah et al. (2021) declared that decisions about the predicting severity of the disease should be based on clinical findings such as age, comorbidities, and laboratory parameters, including the absolute lymphocyte count, C-reactive protein levels, and D-Dimer levels rather than Ct value [41]. On the other hand, He et al. (2020) concluded that to recompense the potential risk of false-negative rRT-PCR, chest CT should be used for clinically suspected cases with negative primary RT-PCR [47]. The analysis of our data showed a significant association among Ct values and some comorbidities such as HTN, CKD, malignancy, and hematologic disorders. One of the reasons can be in terms of the age of the patients with low CT which makes the need for medical interventions and supportive measures in the elderly, especially the patients with risk factors. In support of our data, Biswas et al. (2021), in a comprehensive analysis, showed that male patients with COVID-19 were significantly related to increased risk of mortality compared to the female patients [48]. Moreover, it was reported that the patients with age ≥50 years with COVID-19 were significantly associated with increased risk of mortality as compared to the patients with age <50 years. Besides, Biswas et al. revealed that except for liver disease, comorbid conditions such as hypertension, diabetes, kidney disease, respiratory disease, CVD, cerebrovascular disease, and cancer were significantly higher in nonsurvivors compared to survivors [48]. The present study has encountered some limitations, including a lack of some information and variables such as obesity and other complications.

In conclusion, elderly patients with lower Ct and at least one comorbidity and male cases were significantly associated with the increased risk for COVID-19-related mortality. Moreover, the mortality was significantly higher in patients with DM, kidney disease, and respiratory disease. The initial quantification of SARS-CoV-2 is provided as with useful ground to have better prognostic markers in the clinical management, the treatment of disease, and resource allocation.

## Figures and Tables

**Table 1:** Demographic and comorbidity information of suspected patients with COVID-19 in total population, negative and positive SARS-CoV-2 rRT-PCR.

Variable	Total (*n* = 8318) number (%)	PCR negative (*n* = 5068) number (%)	PCR positive (*n* = 3250) number (%)	*p* value
Age				
<1	194 (2.33)	162 (3.2)	32 (1)	**<0.001**
1–14	542 (6.5)	458 (9.1)	84 (2.6)	
15–24	264 (3.2)	194 (3.8)	70 (2.1)	
25–44	1447 (17.4)	771 (15.2)	676 (20.8)	
45–64	2740 (33)	1489 (29.4)	1251 (38.6)	
≥65	3123 (37.6)	1990 (39.3)	1133 (34.9)	

Sex				
Male	4287 (51.5)	2655 (52.4)	1632 (50.2)	0.053
Female	4031 (48.5)	2413 (47.6)	1618 (49.8)	

Outcome				
Discharge	7679 (92.3)	4764 (94)	2915 (89.7)	**<0.001**
Death	639 (7.7)	304 (6)	335 (10.3)	

Comorbidity				
Cardiovascular disease	2818 (33.9)	1779 (35.1)	1039 (32)	**0.003**
Diabetes	2140 (25.7)	1244 (24.6)	896 (27.6)	**0.002**
Hypertension	1026 (12.3)	579 (11.4)	447 (13.8)	**0.002**
Brain and neurologic disorder	576 (6.9)	434 (8.6)	142 (4.4)	**<0.001**
Kidney diseases	516 (6.2)	384 (7.6)	132 (4.1)	**<0.001**
Malignancy	515 (6.2)	416 (8.2)	99 (3.1)	**<0.001**
Respiratory disorder	356 (4.3)	251 (4.6)	105 (3.2)	**<0.001**
GI diseases	168 (2)	129 (2.6)	39 (1.2)	**<0.001**
Blood disorder	125 (1.5)	99 (2)	26 (0.8)	**<0.001**
Liver disease	90 (1.1)	65 (1.3)	25 (0.8)	**0.027**
Pregnancy	80 (1)	48 (1)	32 (1)	0.86
Others	173 (2.1)	95 (1.9)	78 (2.4)	0.1
No comorbidity	3216 (38.7)	1805 (35.6)	1411 (43.4)	**<0.001**
≥1 comorbidity	5102 (61.3)	3263 (64.4)	1839 (56.6)	

*P* values of < 0.05 were considered statistically significant, and it is shown in bold.

**Table 2:** Demographic and comorbidity information of patients with positive SARS-CoV-2 rRT-PCR based on cycle threshold value (Ct).

Variable	Number (%) of cycle threshold value (Ct)			*p* value
	A (*n* = 336)	B (*n* = 2464)	C (*n* = 451)	
Age				
<1	2 (0.6)	15 (0.6)	15 (3.3)	**<0.001**
1–14	14 (4.2)	41 (1.7)	29 (6.4)	
15–24	10 (3)	48 (2)	12 (2.7)	
25–44	67 (19.9)	535 (21.8)	74 (16.4)	
45–64	110 (32.7)	998 (40.6)	144 (31.9)	
≥65	133 (39.6)	823 (33.4)	177 (39.2)	

Sex				
Male	159 (47.3)	1250 (50.7)	224 (49.7)	0.48
Female	177 (52.7)	1214 (49.3)	227 (50.3)	

Outcome				
Discharge	280 (83.3)	2221 (90.1)	415 (92)	**<0.001**
Death	56 (16.7)	243 (9.9)	36 (8)	

Comorbidity				
Cardiovascular disease	125 (37)	765 (31.1)	149 (33)	0.06
Diabetes	110 (32.7)	664 (27)	122 (27)	0.08
Hypertension	63 (18.8)	322 (13.1)	62 (13.8)	**0.018**
Brain and neurologic disorder	16 (4.8)	104 (4.2)	22 (4.9)	0.7
Kidney diseases	24 (7.1)	83 (3.4)	25 (5.5)	**0.001**
Malignancy	12 (3.6)	60 (2.4)	27 (6)	**<0.001**
Respiratory disorder	9 (2.7)	81 (3.3)	15 (3.3)	0.83
GI diseases	3 (0.9)	29 (1.2)	7 (1.6)	0.68
Blood disorder	4 (1.2)	14 (0.6)	8 (1.8)	**0.02**
Liver disease	2 (0.6)	19 (0.8)	4 (0.9)	0.89
Pregnancy	3(0.9)	23(0.9)	6 (1.3)	0.7
Others	8 (2.4)	61 (2.5)	9 (2)	0.8
No comorbidity	126 (8.9)	1103 (78.1)	183 (13)	**0.01**
≥ 1 comorbidity	210 (11.4)	1361 (74)	268 (14.6)	

*P* values of < 0.05 were considered statistically significant, and it is shown in bold. A: Ct 10–20; B: Ct 21–30; C: Ct 31–40.

**Table 3:** Demographic and comorbidity information of patients with positive SARS-CoV-2 rRT-PCR based on discharge and death.

Variable	Total (*N* = 3246^*∗*^) number (%)	Discharged (*N* = 2911) number (%)	Death (*N* = 335) number (%)	*p* value
Age				
<1	32 (1)	31 (1.06)	1 (0.3)	<0.001
1–14	84 (2.6)	84 (2.9)	0 (0.0)	
15–24	70 (2.1)	69 (2.4)	1 (0.3)	
25–44	676 (20.8)	648 (22.3)	28 (8.4)	
45–64	1251 (38.6)	1142 (39.2)	109 (32.5)	
≥65	1133 (34.9)	937 (32.2)	196 (58.5)	

Sex				
Male	1632 (50.2)	1433 (49.2)	199 (59.4)	**<0.001**
Female	1618 (49.8)	1482 (50.8)	136 (40.6)	

Comorbidity				
Cardiovascular disease	1039 (32)	919 (31.5)	120 (35.8)	0.1
Kidney diseases	132 (4.1)	111 (3.8)	21 (6.3)	**0.031**
Diabetes	896 (27.6)	779 (26.7)	117 (34.9)	**0.001**
Hypertension	447 (13.8)	408 (14)	39 (11.6)	0.23
Malignancy	99 (3)	86 (3)	13 (3.9)	0.35
Respiratory disorder	105 (3.2)	86 (3)	19 (5.7)	**0.008**
Liver disease	25 (0.8)	21 (0.7)	4 (1.2)	0.35
GI diseases	39 (1.2)	35 (1.2)	4 (1.2)	0.9
Blood disorder	26 (0.8)	25 (0.9)	1 (0.3)	0.27
Brain and neurologic disorder	142 (4.4)	125 (4.3)	17 (5.1)	0.5
Pregnancy	32 (1.1)	31 (1.06)	1 (0.3)	0.17
Others	78 (2.4)	71 (2.5)	7 (2.1)	0.6
No comorbidity	1411 (43.4)	1298 (44.5)	113 (33.7)	**<0.001**
Comorbidity	1839 (56.6)	1617 (55.5)	222 (66.3)	

*P* values of < 0.05 were considered statistically significant, and it is shown in bold.

**Table 4:** Demographic and comorbidity information of patients with positive SARS-CoV-2 rRT-PCR based on sex.

Variable	Total (*N* = 3246)^*∗*^ number (%)	Female (*N* = 1615) number (%)	Male (*N* = 1631) number (%)	*p* value
Age				
<1	32 (1)	12 (0.7)	20 (1.2)	**0.008**
1–14	84 (2.6)	36 (2.2)	48 (2.9)	
15–24	70 (2.1)	40 (2.5)	30 (1.8)	
25–44	676 (20.8)	339 (21)	337 (20.6)	
45–64	1251 (38.6)	663 (41)	588 (36)	
≥65	1133 (34.9)	525 (32.5)	608 (37.3)	

Outcome				
Discharge	2915 (89.7)	1482 (91.6)	1433 (87.8)	**<0.001**
Death	335 (10.3)	136 (8.4)	199 (12.2)	

Variable				
Cardiovascular disease	1039 (32)	554 (34.2)	485 (29.7)	**0.006**
Diabetes	896 (27.6)	518 (32)	378 (23.2)	**<0.001**
Hypertension	447 (13.8)	259 (16)	188 (11.5)	**<0.001**
Brain and neurologic disorder	142 (4.4)	74 (4.6)	68 (4.2)	0.57
Kidney diseases	132 (4.1)	63 (3.9)	69 (4.2)	0.62
Respiratory disorder	105 (3.2)	52 (3.2)	53 (3.2)	0.95
Malignancy	99 (3)	53 (3.3)	46 (2.8)	0.44
GI diseases	39 (1.2)	23 (1.4)	16 (1)	0.24
Blood disorder	26 (0.8)	13 (0.8)	13 (0.8)	0.98
Liver disease	25 (0.8)	15 (0.9)	10 (0.6)	0.3
Others	78 (2.4)	50 (3.1)	28 (1.7)	**<0.001**
Comorbidity	1839 (56.6)	979 (60.5)	860 (52.7)	**<0.001**
No comorbidity	1411 (43.4)	639 (39.5)	772 (47.3)	

^*∗*^Age of 4 cases, unknown. *P* values of < 0.05 were considered statistically significant, and it is shown in bold.

## Data Availability

Data are available on request from the authors.
